# Comparison of Changes in Biochemical Markers for Skeletal Muscles, Hepatic Metabolism, and Renal Function after Three Types of Long-distance Running: Observational Study

**DOI:** 10.1097/MD.0000000000003657

**Published:** 2016-05-20

**Authors:** Kyung-A Shin, Ki Deok Park, Jaeki Ahn, Yongbum Park, Young-Joo Kim

**Affiliations:** From the Department of Clinical Laboratory Science (K-AS), Shinsung University, Chungnam; Department of Rehabilitation Medicine (KDP), Gachon University Gil Medical Center, Incheon; and Department of Rehabilitation Medicine (JA, YP, Y-JK), College of Medicine, Sanggye-Paik Hospital, Inje University, Seoul, Republic of Korea.

## Abstract

The purpose of this study is to compare changes in biochemical markers for the skeletal muscles, hepatic metabolism, and renal function based on extreme long-distance running.

Among healthy amateur endurance athletes who participated in a marathon, 100 km-, or 308 km ultramarathon, 15 athletes with similar physical and demographic characteristics were chosen to be the subjects in this study, upon completion of each course. The subjects’ blood was collected before and after the course to identify biochemical markers for the skeletal muscles, hepatic metabolism, and renal function.

After all of the courses, creatinine kinase (CK), lactate dehydrogenase (LDH), aspartate aminotransferase (AST), alanine transaminase (ALT), blood urea nitrogen (BUN), and creatinine were found to be significantly increased compared with values obtained before the race (*P* <0.05 for each marker). CK, LDH, AST, and LDH were significantly higher after completion of the 100 km race than the marathon (*P* <0.05) and were significantly higher after the 308 km race than the marathon or 100 km race (*P* <0.05). Total protein was significantly lower after the 308 km race than the marathon or 100 km race (*P* <0.05). Albumin significantly increased after the marathon but significantly decreased after the 308 km course (*P* <0.05). Total and direct bilirubin were significantly increased after the 100 km and 308 km races (*P* <0.05), and were significantly higher after the 308 km than the marathon or 100 km course (*P* <0.05). BUN was significantly higher after the 100 km race than the marathon (*P* <0.05) and was significantly lower after the 308 km than the 100 km race (*P* <0.05). Creatinine was significantly higher after the marathon and 100 km than the 308 km race (*P* <0.05). Uric acid significantly increased after the marathon and 100 km race (*P* <0.05); it was significantly higher after completing the marathon and 100 km than the 308 km race (*P* <0.05).

Muscular damage, decline in hepatic function, and hemolysis in the blood were higher after running a 308 km race, which is low-intensity running compared with a marathon, and a temporary decline in renal function was higher after completing a 100 km race, which is medium-to-high intensity.

## INTRODUCTION

Appropriate exercise is effective in health promotion and prevention of disease, such as hypertension, hyperlipidemia, and diabetes, however inadequate or extreme exercise over threshold could lead to increased risk of cardiovascular disease and all-cause mortality.^1,2^

Endurance exercise induces cellular changes within the body, increasing cytokine levels and bringing about changes in the muscle, cartilage, heart, liver, and kidneys.^3–9^ Muscles are damaged as a result of metabolic and mechanical factions caused by intense, long-time exercise.^10^ Muscle injury induces rhabdomyolysis via structural damage to the myocytes and protein leakage.^11–13^ Rhabdomyolysis is a form of damage to the skeletal muscles, most commonly caused by trauma.^14^ Recently, studies have found that atraumatic rhabdomyolysis occurs in healthy persons after extreme exercise.^13^ For instance, exertional rhabdomyolysis without symptoms can be caused by running a 246 km ultramarathon.^13^

Increases in serum enzymes that are isolated within the blood, such as creatinine kinase (CK) and lactate dehydrogenase (LDH), serve as markers of acute or chronic muscular damage and cell necrosis.^10^ Activation of CK and LDH may increase symptoms such as pain, fatigue, and a decline in muscular strength during high-intensity, long-distance exercise because of damaged skeletal muscles.^13,15,16^ Noakes^17^ reported that the increase in CK after long-duration exercise is more closely related to the duration of exercise than the intensity.

Alanine transaminase (ALT) and aspartate aminotransferase (AST) serve as markers of liver disease, and increases in AST, ALT, and LDH after long-distance exercise such as an ultramarathon induces chronic liver injury.^18–20^ In particular, ALT and γ-GTP serve as specific markers for liver injury, and their levels are increased after long-distance running.^3,21–23^ Meanwhile, long-distance running is reported to induce not only liver injury but also temporary acute kidney injury.^9^

However, such reports estimate changes in biochemical markers after a single type of long-distance running, and there are few studies examining differences in biochemical markers based on distance.

In this context, this study sought to assess changes in biochemical markers related to skeletal muscles, hepatic metabolism, and renal function before and after long-distance running of three distances: marathon, 100 km, and 308 km.

## MATERIALS AND METHODS

### Subjects and Exercise Protocol

The subjects of this study were chosen from a sample of male volunteers. The subjects in the marathon group were required to have finished a full marathon at least five times. Those in the 100 km ultramarathon group were required to have finished a 100 km ultramarathon at least twice, and those in the 308 km ultramarathon group were required to have finished at least one 308 km ultramarathon. The subjects in this study had to finish a marathon within 4 hours and 30 minutes, a 100 km ultramarathon within 15 hours, or a 308 km ultramarathon within 64 hours. Overall, 23 out of 27 volunteers finished the marathon, 20 out of 25 volunteers finished the 100 km ultramarathon, and 18 out of 23 finished the 308 km ultramarathon. Among those who finished, 15 whose personal characteristics, such as age, body mass index, exercise career, and VO_2max_, were similar were chosen for further study. The subjects’ blood was collected 2 hours before the race (resting baseline) and immediately after the run. The purpose and procedures of this study were explained to all participants, and all provided written consent. This study excluded those individuals who failed to finish the course, failed to finish the course within the prescribed time, had a resting blood pressure ≥140/90 mm Hg, had exercise-induced hypertension (<140 mm Hg resting systolic blood pressure (SBP) and ≥250 mm Hg maximum exercise SBP), or were taking medication for cardiovascular disease, diabetes, renal disease, hepatic disease, or high blood pressure. When the courses started, the temperature and humidity of the marathon, 100 km ultramarathon, and 308 km ultramarathon were 20°C and 47%, 23°C and 50%, and 22°C and 40%, respectively. The data detailing the subjects’ personal careers were collected using questionnaires (Table 1).

**TABLE 1:**
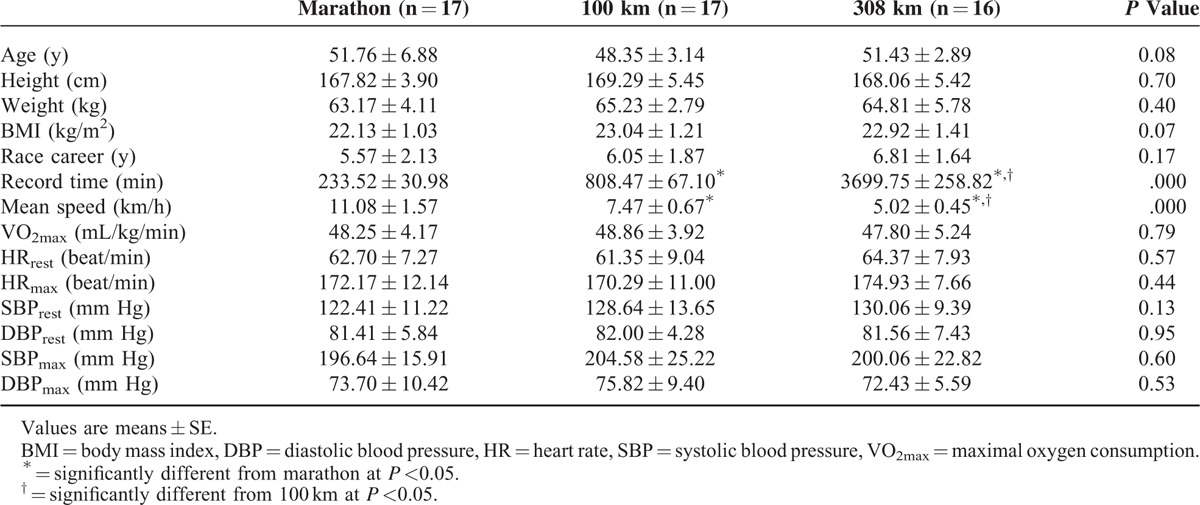
Characteristics of Demographics and Cardiorespiratory Fitness in Study Participants

### Graded Exercise Testing

The Bruce protocol was used for the exercise tolerance test. The test utilized a 12-channel real-time electrocardiograph Q4500 (Quinton instrument company, Washington, US), a respiratory gas analyzer QMC (Quinton instrument company, Washington, US), an automated blood pressure monitor and pulse oximeter (Model 412), and a treadmill Medtrack ST 55 (Quinton instrument company, Washington, US). We calibrated treadmill before graded exercise testing and the subjects had to do hamstring stretching exercise for the prevention of injury and treadmill adaptation.^24,25^ The test was ended when the participant reported subjective symptoms such as chest pain or dizziness or with the occurrence of dangerous cardiac events, and abnormal blood pressure responses on the monitors, based on the guidelines of the American Heart Association (ACC/AHA).^26^

The resting heart rate and blood pressure were measured before the exercise tolerance test, and the heart rate, blood pressure, rating of perceived exertion (RPE), respiratory exchange ratio, and oxygen consumption (VO_2_) were recorded at 1 minute before each step. The 6 to 20 Borg scale was used to measure the RPE; the RPE was explained to the participants before the exercise tolerance test began and they were asked to immediately state their perceived exertion according to the Borg's scale every time the difficulty was changed.^27^

### Blood Sampling

Blood was collected from the antecubital vein to assess changes in the blood biochemical elements before and after the marathon, 100 km-, and 308 km ultramarathon, using the criteria found in the standardized guidelines of the Clinical and Laboratory Standards Institute (CLSI). The blood was collected into vacuum collecting tubes BD Vacutainer (Becton, New Jersy, US) and was separated from the serum at 3400 rpm for 10 minutes for immediate analysis. T(total)-protein, albumin, AST (also known as glutamic oxaloacetic transaminase (GOT)), ALT (also known as glutamate-pyruvate transaminase (GPT)), γ-GTP, alkaline phosphatase (ALP), T-bilirubin, D(direct)-bilirubin, CK, LDH, blood urea nitrogen (BUN), creatinine, and uric acid were measured using a Toshiba TBA-200FR Neo analyzer (Toshiba Medical Systems, Japan). Regarding the analysis methods, AST and ALT were assessed using the GSCC method, creatinine the Jaffe reaction method, BUN the urease glutamate dehydrogenase method, CK the Japan Society of Clinical Chemistry method, LDH the UV-rate method, T-protein the Biuret method, and albumin was assessed using the bromcresol green method. The same examiner analyzed all blood samples using identical devices and examination methods.

### Statistical Analysis

The data are presented as the mean and standard deviation. The general characteristics of the participants and the results of the exercise tolerance test were analyzed using one-way ANOVA, and the Bonferroni correction was used for the *a posteriori* tests. Two-way repeated measures ANOVA was used to verify the interaction effects of each item with time (before and after the course). When there was an interaction effect, a paired *t* test was used to analyze the differences before and after the course. One-way ANOVA was used to identify differences among the groups, and the Bonferroni correction was used for the *a posteriori* tests. The significance level for the results was set at *P* <0.05, and SPSS version 18.0 was used for statistical analysis.

## RESULTS

Table 2 shows the hepatic metabolism results based on the running distance. T-protein and ALP did not show significant effects of time or group. Albumin was significantly increased after the marathon but significantly decreased after the 308 km ultramarathon (*P* <0.05 for each). It was significantly lower after completing the 308 km than the marathon or 100 km course. T-bilirubin was significantly increased after the 100 km and 308 km courses (*P* <0.05) and was significantly higher after the 308 km race than the marathon or 100 km course (*P* <0.05). D-bilirubin was significantly increased after the 100 km and 308 km courses (*P* <0.05) and was significantly higher after the 100 km course than the marathon (*P* <0.05) and higher after the 308 km race than the marathon or 100 km race (*P* <0.05). AST and ALT increased significantly after completion for all of the courses, compared with before all courses (*P* <0.05); these markers were significantly higher after the 100 km run than the marathon (*P* <0.05) and were significantly higher after the 308 km run than the marathon or 100 km (*P* <0.05). The γ-GTP level was significantly increased after the 100 km and decreased after the 308 km course (*P* <0.05) and was significantly lower after the 308 km race than the marathon (*P* <0.05).

**TABLE 2:**
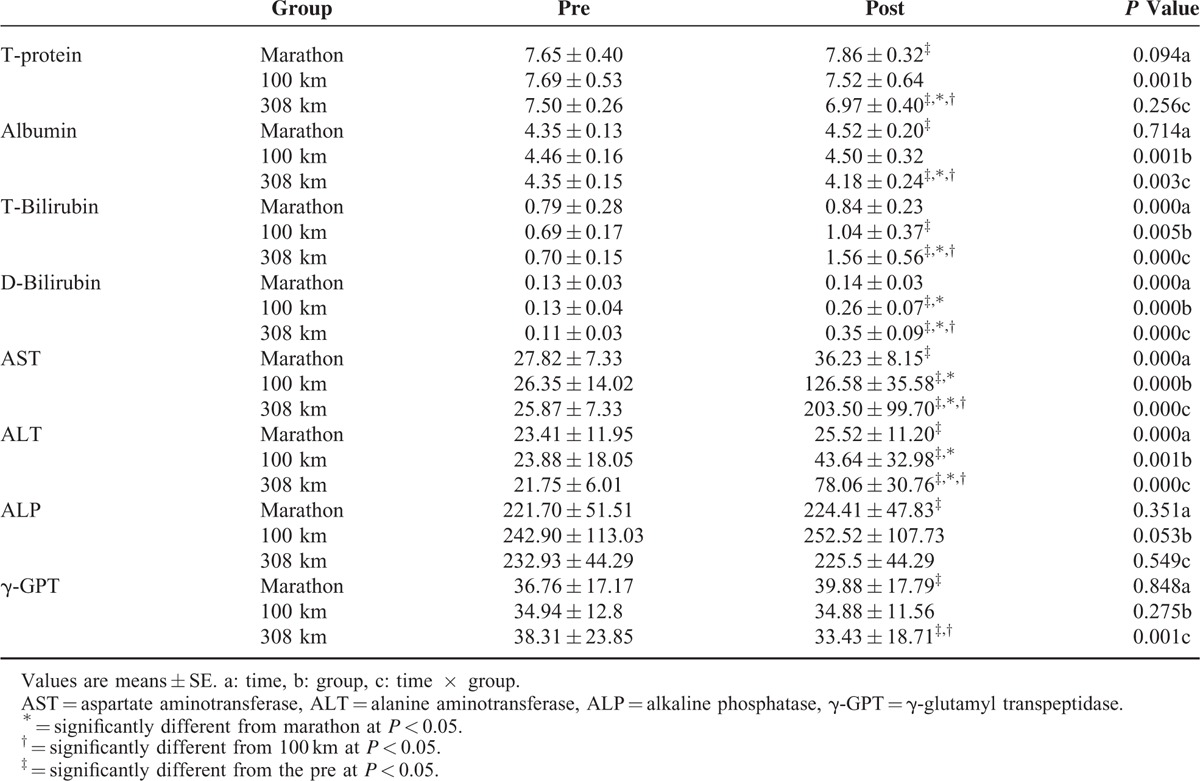
Changes in Hepatic Metabolism based on Running Distance

Table 3 shows the changes in the markers of renal function and muscle metabolism. BUN was significantly increased after all courses (*P* <0.05); it was significantly higher after completion of the 100 km race than the marathon (*P* <0.05) and was significantly lower after completion of the 308 km than the 100 km race (*P* <0.05). Creatinine was significantly increased after all courses (*P* <0.05); it was significantly higher after the marathon and 100 km than the 308 km race (*P* <0.05). Uric acid was significantly increased after the marathon and 100 km courses, but not the 308 km course (*P* <0.05); it was significantly higher after the marathon and 100 km race than the 308 km (*P* <0.05). CK and LDH increased significantly after completion of all courses (*P* <0.001); these markers were significantly higher after the 100 km race than the marathon and were significantly higher after the 308 km course than the marathon or 100 km course (*P* <0.05).

**TABLE 3:**
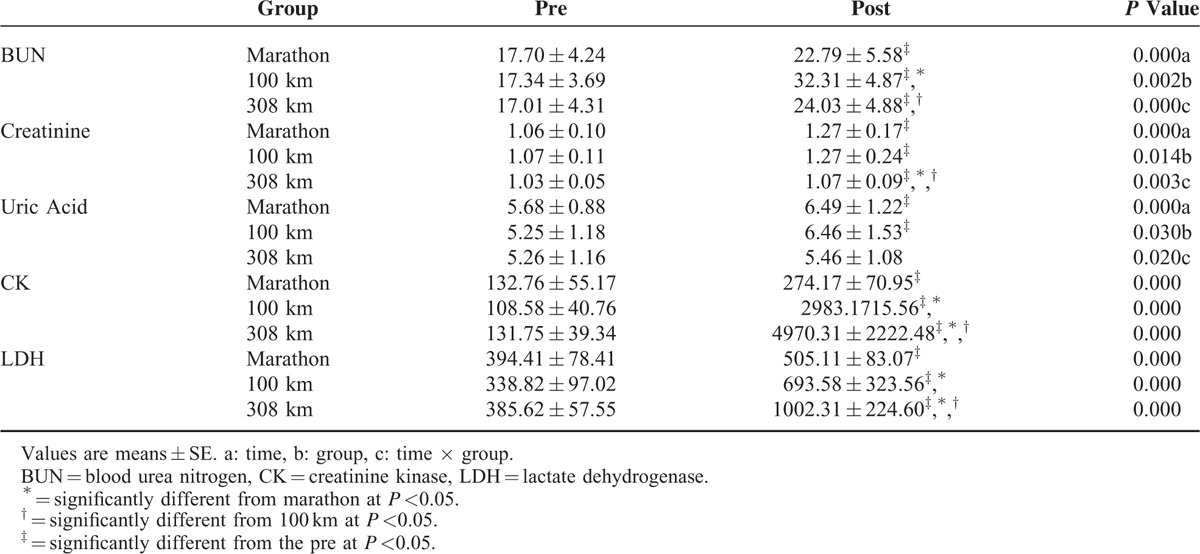
Changes in Markers for Renal Function and Muscular Injury Based on Running Distance

## DISCUSSION

This study aimed to identify changes in biochemical markers of the skeletal muscles, liver, and kidney after completion of a marathon, 100 km-, and 308 km ultramarathon.

Completing a marathon involves weight-bearing and long-duration running, thus inducing changes in the heart and skeletal muscles caused by repetitive muscle contraction.^2,28^ Muscular injury is accompanied by atraumatic rhabdomyolysis caused by structural damage and protein leakage in the myofibrils, acute inflammatory response, and a decline in muscular strength.^13,29–31^ For instance, exertional rhabdomyolysis without symptoms can be caused by running a 246 km ultramarathon.^13^ CK, LDH, and myoglobin may serve as markers for assessing the degree of muscular injury; increases in these enzymes reflect skeletal muscle disability because of cell necrosis and muscular damage.^32^

Skenderi et al^13^ found that muscle damage was caused by high-intensity, long-distance exercise because of significant increases in serum CK and LDH during a 246 km ultramarathon.

Degrees of muscular injury induced by long-distance running may be different based on physical fitness level, marathon distance, and exercise intensity.^10,17^ Kim et al^33^ reported that a long-distance marathon induces greater muscular injury, as the blood CK increased at least 10 folds after a 200 km ultramarathon than after a marathon.

The results of this study showed that CK and LDH increased after the marathon, and the range of the increase was higher after the 308 km ultramarathon, that is, the low-intensity, long-distance running. In particular, the increase in CK levels after the 308 km ultramarathon was 20 folds greater than that after the marathon, and 1.4 folds greater than that after the 100 km ultramarathon. This supports other studies that report that the increase in CK after the 308 km ultramarathon is more closely related to exercise duration than to physical fitness level or exercise intensity.^10,17^ Furthermore, higher CK and LDH during a long-distance ultramarathon than a marathon may be caused by the accumulation of fatigue because of energy exhaustion and changes in energy metabolism.

Marathon running induces not only musculoskeletal injury but also atraumatic, temporary dysfunction and injury to the cardiovascular system, liver, and kidney.^3,4,6,7,9^ Increases in AST, ALT, and LDH after a 24-hour ultramarathon indicate chronic liver injury because of long-time extreme exercise.^20^

Because LDH and AST are found both in the liver and muscle cells, increases in these enzymes may not be a clear marker for liver cell damage.^5^ However, ALT and γ-GTP are more specific markers for liver injury.^22,23^

The data assessing liver injury using enzymes such as LDH, ALT, AST, and γ-GTP during an ultramarathon are controversial.^5^ According to Skenderi et al^13^, ALT significantly increased after a 246 km ultramarathon, but the rate of increase was not higher than that of other enzymes, indicating that the skeletal muscles were more damaged than the liver. Also, the γ-GTP level did not change after the race, indicating that the increases in other variables are likely caused by injury in the muscle cells rather than liver injury.^13^ Soeder et al^34^ reported that γ-GTP decreased immediately after a marathon and ALT and γ-GTP did not change after a half marathon, suggesting that the increase may be caused by muscular rather than liver injury.^35^

Meanwhile, incremental increases in liver enzymes such as AST, ALT, and γ-GTP after short-distance and long-distance ultramarathons indicate injury in the liver and muscle cells,^3,20,36^ and the degree of liver injury is proportional to the work load.^20^

In this study, AST and ALT increased significantly after completion of all courses, compared with the baseline taken before the race, and increased as the running distance increased. Meanwhile, ALT, specific for the liver, showed a smaller increase compared with AST. γ-GTP increased after marathon completion but decreased after finishing the 308 km course. T-protein decreased after the 308 km course; this decrease is caused by a decline in albumin and reflects protein metabolism in the liver and impaired aerobic function in the liver cells.^20^

The decline in hepatic function caused by running the ultramarathon is related to changes in the liver cell membrane by lipid peroxidation because of impaired blood flow and the release of free radicals.^37–40^ This suggests that the liver undergoes a temporary decline in function during long-distance rather than short-distance running.

The increase in bilirubin after long-distance running is caused by hemocytocatheresis, physiological turnover, and catabolism of hemoglobin, or by liver injury.^30,40^ In particular, “foot strike hemolysis” has been found to occur in long-distance runners because of mechanical injury related to running, and such hemolysis within vessels is induced as a side effect of endurance exercise.^5,41,42^

Wu et al^20^ reported that T-bilirubin and D-bilirubin increased immediately after a 24-hour ultramarathon, and this increase was related to hemolysis because of long-distance running. In the present study, T-bilirubin and D-bilirubin increased after all courses, and increased 1.5 folds more after the 100 km than the marathon and 2 folds more after the 308 km than the marathon. Such results are caused by increases in intramuscular hemocytocatheresis during long-distance running, partial disability of hepatic metabolism, and hemolysis.^35,43^

Renal dysfunction can be measured by BUN and creatinine.^32^ Blood BUN and creatinine increase after marathon completion.^9^ In this study, BUN and creatinine increased by more than the upper reference limit (URL) after the marathon and 100 km course. In particular, BUN increased 1.4-folds more after the 100 km race than the marathon and the 308 km race, indicating that the temporary decline in renal function was greater because of the decline in renal blood flow.

The exercise intensity of a marathon is considered high-intensity (average 11.3 km/h), whereas 100 km running is medium-to-high intensity (average 7.4 km/h) and 308 km running is low intensity (4.9 km/h).^44^ Thus, the increase in BUN by more than the URL after the 100 km race may be a result of the prolonged accumulation of nonprotein nitrogen in the blood during long-duration running (10–15 hours) with medium-to-high intensity. However, to identify acute kidney injury, measurements of markers such as neutrophil gelatinase-associated lipocalin (NGAL) or kidney injury molecule-1 (KIM-1) are needed. Although uric acid significantly increased after the marathon and 100 km, the range of the increase was less than the URL, and the clinical significance of this increase may be slight.

A limitation of this study is that while the subjects who participated in the marathon, 100 km- or 308 km ultramarathon had similar personal characteristics, the meals, and beverages offered to them, as well as the starting time of the competitions, differed. Additionally, we could not control environmental factors such as the temperature and humidity, and we could not compare changes in biochemical variables during the recovery period.

## CONCLUSIONS

In conclusion, muscular injury and decline in hepatic function because of increases in the leakage of organ-specific enzymes into the blood were higher after completion of the 308 km course (low-intensity long-distance running) compared with the marathon or 100 km ultramarathon. In addition, hemolysis in the blood was higher after the 308 km race than the marathon or the 100 km race, and the temporary decline in renal function because of a decline in renal blood flow was highest after the 100 km race (medium- to high-intensity running).
